# Who Accommodates Whom? Bidirectional Linguistic Accommodation and Progressive Interpersonal Convergence in Human–AI Conversations

**DOI:** 10.3390/bs16050720

**Published:** 2026-05-07

**Authors:** Pengbo Chen, Huining Guan, Eui Jun Jeong

**Affiliations:** 1 Department of Digital Culture and Contents, Graduate School, Konkuk University, Seoul 05029, Republic of Korea; 2 Department of Arts and Cultural Management, Graduate School, Hongik University, Seoul 04066, Republic of Korea

**Keywords:** linguistic accommodation, human-AI interaction, self-presentation, function word convergence, interpersonal orientation

## Abstract

Linguistic accommodation during human–AI interaction has been measured in only one direction at a time, leaving the relative magnitude of each side and the trajectory of within-conversation change unresolved. A symmetric within-versus-between conversation dissociation design applied to 1319 multi-turn English GPT-4o conversations from WildChat measures both user-side and model-side function word adaptation within the same data, revealing two distinct temporal dynamics. The model’s adaptation is front-loaded, with strong initial accommodation at the first turn followed by stabilization, while users converge gradually across subsequent turns on interpersonal pronoun dimensions with no progressive change in topic-related categories. In 500 Switchboard human–human conversations, per-conversation similarity slopes are significantly negative (p=0.022), though the multilevel interaction is marginal (p=0.055). Because the pronoun dimensions on which users converge are the primary linguistic markers through which personality traits manifest in natural language use, this progressive convergence may represent a linguistic indicator of shifts in communicative self-presentation during extended human–AI conversation.

## 1. Introduction

Large language models (LLMs) now serve as conversational partners for hundreds of millions of users daily, producing output that is stylistically distinctive in its formal structure, lexical precision, and syntactic regularity (Guo et al., 2025). Communication Accommodation Theory (CAT) predicts that interlocutors adjust their communicative behavior toward or away from their partners during conversation (Giles, 2016; Giles et al., 1991), and the interactive alignment model provides a complementary mechanistic account in which priming at lexical, syntactic, and semantic levels causes interlocutors’ representations to converge during dialogue (Chartrand & Bargh, 1999; Garrod & Pickering, 2004; Pickering & Garrod, 2004). Function words (pronouns, articles, prepositions) reflect interpersonal orientation and self-presentational style rather than topic content (Ireland et al., 2011; Pennebaker et al., 2003); they are also the primary linguistic markers through which personality dimensions manifest in natural language use (Pennebaker et al., 2003). Shifts in function word usage during human–LLM conversation may therefore provide evidence about changes in users’ communicative self-presentation, particularly on the personality-linked dimensions that pronoun distributions are known to express.

Each direction of adaptation has been studied in isolation. Blevins et al. (2026) demonstrated that LLMs strongly converge toward users’ linguistic style across sixteen language models, and Kandra et al. (2025) showed that LLMs syntactically adapt to their conversational partners using a within-versus-between baseline design. On the user side, Zhang and Yu (2025) documented a static register shift at conversation onset without tracking within-conversation trajectories. Population-level evidence indicates that LLM-characteristic vocabulary has already permeated human writing (Liang et al., 2025; Yakura et al., 2024), and recent findings suggest that LLM influence extends to individual self-concept (Li et al., 2026), compounding the population-level effects already documented (Padmakumar & He, 2024; Sourati et al., 2026).

An identification problem remains. If a within-versus-between design measures only user-side similarity (user turn *N* vs. assistant turn N−1), a positive result is ambiguous: it could reflect user-side accommodation, model-side accommodation (the model has already adapted to this user, making the within-conversation assistant turn inherently more similar), or both. Chang and Wang (2025) examined bidirectional accommodation using LSM scores across cultures but without the within-versus-between baseline control that distinguishes conversation-specific adaptation from population-level similarity. Disentangling the two directions requires measuring both in the same data. The distinction is not only quantitative but mechanistic: model-side similarity is a product of conditional text generation (the model’s output distribution is conditioned on the user’s prior turns), whereas user-side convergence, if present, may reflect social–cognitive accommodation or automatic priming operating on a human speaker. A symmetric design that separately characterizes the trajectory of each side can locate the progressive signal and determine whether it originates from the user, the model, or both.

The present study applies a symmetric within-versus-between conversation dissociation design (Danescu-Niculescu-Mizil & Lee, 2011; Danescu-Niculescu-Mizil et al., 2012) to 1319 English GPT-4o conversations from WildChat (Zhao et al., 2024), computing the within-versus-between dissociation in both directions: (1) user-side, comparing user turn *N* to assistant turn N−1, and (2) model-side, comparing assistant turn *N* to user turn N−1. Both directions use the same multilevel model framework, the same between-conversation sampling procedure, and the same linguistic indicators. Aggregate function word similarity is decomposed into its 10 constituent categories to determine which word classes drive the static and progressive effects. These patterns are compared against 500 conversations from the Switchboard corpus (Godfrey et al., 1992) as a human–human baseline, with additional analyses of topic moderation, cross-model consistency (GPT-3.5), turn attrition, and survivorship.

## 2. Related Work

### 2.1. Communication Accommodation and Interactive Alignment

Communication Accommodation Theory (CAT) holds that speakers adjust their communicative behavior toward (convergence) or away from (divergence) their interlocutors, driven by social goals including affiliation, identity maintenance, and communicative efficiency (Giles, 2016; Giles et al., 1991). Convergence has been documented across phonological (Pardo, 2006), lexical (Brennan & Clark, 1996; Dale & Spivey, 2006), syntactic (Bock, 1986; Branigan et al., 2000), and semantic levels (Wynn et al., 2025), and is modulated by social context: speakers converge more toward desired affiliates and may diverge from outgroup members (Giles et al., 1991; Gnisci, 2005). The interactive alignment model (Garrod & Pickering, 2004; Pickering & Garrod, 2004) provides a complementary mechanistic account in which priming at lexical, syntactic, semantic, and situational levels causes interlocutors’ representations to align largely without conscious awareness. Syntactic persistence operates even in the absence of semantic or pragmatic pressure (Bock, 1986), strengthens under lexical overlap (Branigan et al., 2000), and predicts joint decision-making performance when measured as coupled oscillatory dynamics (Fusaroli et al., 2012). These two accounts differ in what drives convergence (social motivation vs. automatic priming), and the distinction matters for human–AI interaction, where the non-human interlocutor lacks social motivation yet may still trigger priming in users.

Partner-specificity provides the empirical anchor for the present design. Brennan and Clark (1996) showed that conversational partners form temporary referential agreements that persist within a conversation but do not transfer to new partners. This specificity means that within-conversation convergence reflects conversation-specific adaptation rather than general stylistic drift, motivating the within-versus-between dissociation design: if users form analogous patterns with LLMs, within-conversation similarity should exceed between-conversation similarity.

Function words are the appropriate indicator for this test because they reflect the interpersonal and self-presentational dimensions of communicative style established in Section 1 (Niederhoffer & Pennebaker, 2002; Pennebaker et al., 2003). Linguistic style matching (LSM) based on function word rates predicts relationship initiation (Ireland et al., 2011), group cohesion (Gonzales et al., 2010), and power dynamics (Danescu-Niculescu-Mizil et al., 2012). The within-versus-between dissociation design follows Danescu-Niculescu-Mizil and Lee (2011), who compared within-conversation coordination against a shuffled baseline to distinguish genuine convergence from population-level similarity, later extended by Doyle et al. (2016) with models robust to cross-domain variation and by Jones et al. (2014) with a normalized accommodation measure controlling for baseline frequency.

### 2.2. Alignment and Divergence in Corpus Studies

Corpus evidence complicates the expectation that convergence is the default outcome of extended dialogue. Reitter and Moore (2014) found positive syntactic priming in Switchboard conversations correlating with task success, and Xu and Reitter (2016a, 2016b) showed convergence in entropy rates and syntactic complexity. Yet Healey et al. (2014) found that interlocutors’ lexical and structural choices diverge over conversation even as mutual understanding improves, arguing that divergence assists in role differentiation and communicative efficiency. Convergence and divergence can therefore coexist across different linguistic levels within the same conversation, a finding directly relevant to the present study’s observation of divergence in Switchboard function words alongside prior convergence findings using different measures in the same corpus.

Fusaroli et al. (2014) formalized this coexistence as a dual process of alignment (convergence on shared representations) and complementarity (differentiation of communicative roles). Duran et al. (2024) provided empirical support: lexical and syntactic alignment decreased over time in collaborative problem solving while semantic alignment increased. Function word divergence in Switchboard may reflect increasing role differentiation (narrator vs. listener) to enhance communicative efficiency. Human–LLM conversations, in which one interlocutor cannot compete for communicative roles, should show a different balance of alignment and complementarity, and the direction of that difference is diagnostic of whether the user-side signal reflects social accommodation or automatic priming.

### 2.3. Human–Computer Linguistic Alignment

The CASA (Computers Are Social Actors) paradigm (Nass et al., 1994; Reeves & Nass, 1996) established that humans apply social rules to computers, including politeness norms and reciprocity. Branigan et al. (2010) extended this to linguistic alignment, finding stronger syntactic alignment with computer interlocutors than with human partners and interpreting the result as reflecting beliefs about interlocutor competence: speakers accommodate more when they believe the interlocutor has limited processing capacity. LLMs complicate this account. Their output is remarkably fluent, which should reduce competence-driven accommodation; yet the CASA effect may operate through social categorization of a non-human agent rather than through specific competence attributions, in which case alignment with LLMs could remain strong. Wade and Roberts (2020) showed that convergence targets both observed and expected linguistic behavior, with convergence amplified when the two are congruent, and Noble and Beinborn (2022) found that alignment strength depends on agent design, perceived agency, and task context. The present data adjudicate between these accounts by measuring whether progressive alignment with a highly fluent AI interlocutor occurs in interpersonal markers (consistent with social–cognitive accommodation) or in structural vocabulary (consistent with competence-driven or topic-driven priming).

### 2.4. User Behavior in Human–LLM Interaction

Zhang and Yu (2025) compared user language in human–LLM versus human–human customer service conversations, finding that users addressing LLM assistants were 14.5% less polite, 5.3% less grammatically fluent, and 1.4% less lexically diverse. Their analysis examined only the first user turn (the initial register shift), leaving within-conversation dynamics unaddressed but predicting the strong static effect ($\beta_{1}$) observed in the present study. On the model side, Blevins et al. (2026) found strong model-side convergence toward users’ linguistic style across sixteen language models, and Kandra et al. (2025) demonstrated syntactic adaptation using a within-versus-between baseline design. Because LLMs trained with RLHF tend to validate user statements and mirror user style (Ouyang et al., 2022; Perez et al., 2023; Sharma et al., 2024; Wei et al., 2024), a unidirectional user-side analysis cannot separate genuine user accommodation from similarity inflated by model-side mirroring. The symmetric bidirectional design (Section 3.2) resolves this confound.

### 2.5. LLM Influence on Human Language, Cognition, and Self-Presentation

Population-level evidence of LLM influence on human language is accumulating. Liang et al. (2025) found that up to 17.5% of computer science paper abstracts showed signs of LLM modification by early 2024, with specific marker words surging in frequency after ChatGPT’s release; Kobak et al. (2025) documented excess frequency ratios of up to 28-fold for LLM-characteristic words (e.g., “delves”) in biomedical abstracts. Yakura et al. (2024) provided causal evidence that LLM interaction reshapes spoken communication patterns, and Guo et al. (2025) established that LLM output is measurably less diverse than human writing across lexical, syntactic, and semantic dimensions. The consequences extend to cognitive diversity: Padmakumar and He (2024) showed that LLM-assisted writing reduces content diversity across users, and Sourati et al. (2026) synthesized evidence of homogenization across linguistic, cognitive, and cultural dimensions.

The link between linguistic accommodation and changes in self-concept measures has been examined experimentally. Li et al. (2026) found that AI-exhibited personality traits can influence users’ self-concept measures during conversation, with the magnitude of alignment increasing with conversation length. Function word usage patterns, particularly pronoun distributions, are among the most reliable linguistic markers of personality traits (Pennebaker et al., 2003). If within-conversation pronoun shifts reflect changes in self-presentational orientation, they may constitute a linguistic pathway through which the self-concept effects documented by Li et al. (2026) operate. Jakesch et al. (2023) found that people cannot reliably distinguish AI-generated from human-written text, removing a potential cognitive barrier to accommodation; Shumailov et al. (2024) showed that recursive AI-to-AI training produces model collapse, suggesting that human-to-AI accommodation and AI-to-human influence may form a feedback loop with cumulative consequences.

LLM output is pragmatically constrained relative to human interlocutors, with limitations in implicature, presupposition, and reference (Ma et al., 2025; Sravanthi et al., 2024) and systematic differences in turn-taking and grounding (Mayor et al., 2025). Users adapting toward such a target are converging on a communicative style that lacks the pragmatic complexity of human conversation, a qualitative asymmetry that shapes the interpretation of any convergence effect and its implications for users’ broader communicative competence.

## 3. Materials and Methods

### 3.1. Within-Versus-Between Dissociation Design

The analytical challenge is distinguishing genuine within-conversation convergence from task-stage effects (both user and assistant language becoming more technical as a task progresses), topic-driven similarity (adjacent turns sharing topic vocabulary), and selection effects (higher-similarity conversations persisting longer, addressed by the survivorship check in Section 4). The paired comparison design addresses the first two confounds.

For each eligible user turn in conversation *c* (all user turns that have a preceding assistant turn, indexed from 1), we compute:

- Within-conversation similarity $S_{\mathrm{within}}\left(N,c\right)$: similarity between user turn *N* and assistant turn N−1 in the same conversation *c*.
- Between-conversation similarity $S_{\mathrm{between}}\left(N,c\right)$: similarity between user turn *N* and a randomly sampled assistant turn from a different conversation $c^{\prime }$, drawn uniformly from the full conversation pool with random seed 42.

Both values are entered into a multilevel model with conversation-level random intercepts:

(1) $$S_{ij}=\beta_{0}+\beta_{1}\cdot {\mathrm{IsWithin}}_{ij}+\beta_{2}\cdot {\mathrm{Turn}}_{ij}+\beta_{3}\cdot {\mathrm{IsWithin}}_{ij}\times {\mathrm{Turn}}_{ij}+u_{0j}+\epsilon_{ij}$$

where *i* indexes observations, *j* indexes conversations, $u_{0j}∼N\left(0,\sigma_{u}^{2}\right)$ is a random intercept for conversation, and ${\mathrm{Turn}}_{ij}$ is centered at the sample mean (mean-centered values are reported in Appendix C).

The four parameters have distinct interpretations. $\beta_{0}$ is the grand intercept (mean between-conversation similarity at the mean turn). $\beta_{1}$ captures the static within-versus-between difference: a positive value indicates that users are more similar to their own interlocutor than to random interlocutors, averaged across turns. $\beta_{2}$ captures the between-conversation trajectory, whether the baseline similarity drifts over turns. A flat between-conversation trajectory ($\beta_{2}\approx 0$) confirms that any change in the within-versus-between gap reflects within-conversation dynamics rather than corpus-level drift. $\beta_{3}$ captures the progressive component: a positive value indicates that the within-versus-between gap widens over turns, meaning users are progressively converging beyond initial register matching.

### 3.2. Symmetric Bidirectional Design

The within-versus-between dissociation is applied in both directions within the same conversations. The user-side analysis (described above) computes similarity between user turn *N* and assistant turn N−1. The model-side analysis reverses the roles: for each assistant turn *N*, we compute (1) within-conversation similarity between assistant turn *N* and the preceding user turn N−1, and (2) between-conversation similarity between assistant turn *N* and a randomly sampled user turn from a different conversation (same sampling algorithm as the unidirectional pipeline, independently implemented; see Section 3.2). The same multilevel model (Equation (1)) is fitted separately for each direction, with “focal turn” replacing “user turn” in the general formulation.

This symmetric design enables direct comparison of the static effect ($\beta_{1}$) and progressive interaction ($\beta_{3}$) across the two directions. If model-side $\beta_{1}$ substantially exceeds user-side $\beta_{1}$, model-side adaptation contributes a larger share of the within-versus-between gap. If the two directions show different progressive dynamics ($\beta_{3}$), this reveals which side drives the over-turn trajectory. The between-conversation baseline provides the same control in both directions: it measures how similar a focal turn is to a random partner turn, establishing the expected similarity absent any conversation-specific adaptation.

Both directions use a harmonized analysis window of focal-turn index 1–10. The first eligible user turn is always index 1 (the first user message has no preceding assistant turn). The first eligible assistant turn is index 0 (the first assistant message does have a preceding user turn), but we exclude it to maintain window symmetry across directions. The model-side turn-0 observation, which reflects the assistant’s initial response to a single user message and exhibits an atypically large within-versus-between gap, is reported separately in Section 4. The user-side and model-side analyses have slightly different observation counts (25,976 vs. 26,000) because conversations may have unequal numbers of user and assistant turns after filtering. Because $\beta_{3}$ estimates are sensitive to between-conversation sampling (a single random partner per within-conversation observation introduces sampling noise into the baseline), we repeated the analysis across 10 different random seeds for both directions; results are reported in Section 4.7.

#### Analysis Pipelines

Two analysis pipelines are used. The unidirectional pipeline (Section 3) was the original implementation, applied to WildChat (full sample and 500-conversation subsample) and to Switchboard for the user-side analysis and syntactic similarity. The symmetric bidirectional pipeline (this section) was independently implemented for the bidirectional comparison on the full 1319-conversation sample. Both pipelines use the same between-conversation sampling algorithm (randomly select a different conversation, then randomly select a partner turn from that conversation), but the two implementations traverse conversations in different orders and maintain independent random-number sequences, producing slightly different observation counts (25,996 vs. 25,976 for the user-side full sample) and different between-conversation baseline draws. The bidirectional pipeline is the primary analysis; the unidirectional pipeline is retained for the Switchboard comparison and for syntactic similarity, where the bidirectional pipeline was not applied. Both pipelines yield directionally consistent $\beta_{3}$ estimates. The difference in significance (p=0.056 unidirectional vs. p=0.003 bidirectional) reflects sampling variation in the between-conversation baseline; the 10-seed robustness analysis (Section 4.7) confirms that $\beta_{3}$ is positive and significant across a wide range of baseline draws.

### 3.3. Per-Conversation Slope Analysis

As a complementary analysis, we compute per-conversation linear slopes of within-conversation similarity across turns and test whether the distribution of slopes differs from zero using one-sample *t*-tests. This analysis answers a different question than the within-versus-between interaction ($\beta_{3}$). The multilevel model tests whether the gap between within- and between-conversation similarity changes over turns (whether users become more similar to their own interlocutor relative to random interlocutors). The per-conversation slope analysis tests whether within-conversation similarity increases over turns without reference to a between-conversation baseline. A positive per-conversation slope indicates increasing absolute similarity to the interlocutor; a positive $\beta_{3}$ indicates increasing relative similarity. The two can dissociate if the between-conversation baseline also changes over turns.

### 3.4. Linguistic Indicators

#### 3.4.1. Function Word Similarity

Following the LIWC tradition (Pennebaker et al., 2001, 2003; Tausczik & Pennebaker, 2010), we compute the rate of words in each of 10 function word categories per turn: first-person singular pronouns, first-person plural, second-person, third-person pronouns, discourse connectives (e.g., “however,” “therefore”), hedges (e.g., “maybe,” “probably”), articles, prepositions, conjunctions, and negations. Full word lists are provided in Appendix A. We represent each turn as a 10-dimensional rate vector and compute cosine similarity between user and partner vectors. Function words are informative for convergence analysis because they reflect communicative style and interpersonal orientation (Ireland et al., 2011; Niederhoffer & Pennebaker, 2002). For the category-level decomposition, per-category similarity is computed as $1-\;|r_{\mathrm{focal}}^{\left(k\right)}-r_{\mathrm{partner}}^{\left(k\right)}|$, where $r^{\left(k\right)}$ is the rate of category *k* in the respective turn, yielding a bounded similarity measure in [0, 1] for each of the 10 categories, analyzed with the same multilevel model specification as the aggregate cosine similarity.

#### 3.4.2. Syntactic Similarity

We parse each turn with spaCy’s en_core_web_sm (v3.8) dependency parser and extract dependency relation patterns as (dependency-label, head-POS, dependent-POS) triples. We compute Jaccard similarity between the user’s pattern set and the partner’s pattern set, capturing structural overlap at the syntactic level independent of specific lexical items. This measure is related to the syntactic priming measures used in prior corpus studies (Branigan et al., 2000; Reitter & Moore, 2014; Xu & Reitter, 2016a) but operates at a coarser level (pattern-type overlap rather than sequential repetition or complexity convergence).

### 3.5. Datasets

#### 3.5.1. WildChat (Human–LLM)

We use the WildChat-4.8M corpus (Zhao et al., 2024), filtering to English-language conversations with GPT-4o models (gpt-4o, gpt-4o-2024-05-13, gpt-4o-2024-08-06, gpt-4o-2024-11-20) containing at least five user-assistant turn pairs (10 messages total). This yields 1319 qualifying conversations with 48,445 turns. We analyze all 1319 conversations for the function word analysis. For the syntactic similarity analysis, which requires dependency parsing of each turn, we analyze a subsample of 500 conversations (random seed 42). The mean tokens per turn are 324 for user messages and 355 for assistant messages (medians 18 and 271, respectively), reflecting the long-form written nature of LLM interaction with a heavy right tail driven by long-pasted prompts and elaborated responses. The five-turn minimum selects for more sustained and engaged interactions. To assess sensitivity, we re-estimated the user-side $\beta_{3}$ at stricter thresholds (minimum 7 and 9 user turns within the analysis window). The $\beta_{3}$ estimate is stable across thresholds (+0.0038, p=0.003 at 1319, 1319, and 1313 conversations, respectively), because nearly all conversations contribute observations across the full turns 1–10 window. The results characterize extended multi-turn human–AI conversations. Users who ask a single question and leave may exhibit different accommodation dynamics.

#### 3.5.2. Switchboard (Human–Human)

We use 2438 conversations from the Switchboard corpus (Godfrey et al., 1992), a collection of spontaneous telephone conversations between strangers on 70 assigned topics (e.g., “child care,” “gun control”). We parse the Mississippi State word-aligned transcriptions, interleave speaker A and B utterances by timestamp, and merge consecutive same-speaker utterances into dialogue turns. We remove silence markers, laughter annotations, and partial-word markers while preserving lexical content. We analyze 500 randomly sampled conversations. The mean tokens per turn is 18 (median 11), reflecting the short, rapid nature of spoken telephone conversation with frequent backchannels.

#### 3.5.3. Cross-Corpus Confounds

The WildChat–Switchboard comparison involves three confounds that prevent causal attribution of the convergence–divergence asymmetry to interlocutor type. First, written and spoken language differ systematically in function word distributions (Biber, 1991; Chafe, 1982), and the mean tokens per turn differ by more than an order of magnitude (324 vs. 18), which affects the stability of function word rate estimates and the baseline similarity level (∼0.55 vs. ∼0.24). Second, all WildChat conversations involve the same interlocutor system (GPT-4o), whereas each Switchboard conversation involves a unique speaker pair, complicating cross-corpus $\beta_{1}$ comparison (Guo et al., 2025). Third, short Switchboard turns (backchannels) may produce degenerate function word vectors. We therefore compare the direction of effects across corpora rather than magnitudes. A written human–human baseline (e.g., online chat) is needed to disambiguate modality from interlocutor effects.

### 3.6. Data Processing

For WildChat, we download parquet shards (shards 40–58 of 86) from HuggingFace and filter using DuckDB for efficiency. For Switchboard, we parse the MS-98 word-aligned transcripts from the locally stored corpus (2438 conversation pairs across 30 directory shards). Between-conversation pairs are sampled uniformly from the full conversation pool (not topic-matched) with one between sample per within pair and random seed 42. All analysis code, model results, and processed similarity data are provided in Dataset S1.

### 3.7. Feature Extraction

Function word rates use whitespace tokenization with lowercase normalization and punctuation removal. Syntactic features use spaCy v3.8.11 with the en_core_web_sm model (NER disabled for speed). All features are computed at the turn level.

### 3.8. Statistical Models

We fit mixed-effects models using statsmodels.mixedlm (Seabold & Perktold, 2010) in Python 3.12 with REML estimation and Powell optimization. All random-intercept models converged without warnings. The Turn variable is centered at the sample mean. We limit analyses to turns 1–10 to focus on the early-conversation trajectory and avoid sparse data at later positions. We report raw coefficients throughout. For interpretation, a $\beta_{3}$ coefficient on the order of $10^{-3}$ per turn in cosine similarity corresponds to a small effect by conventional benchmarks; category-level effect sizes are reported in Section 4.

Random effects include a random intercept for conversation ID only (Raudenbush & Bryk, 2002). Random-slope specifications and alternative inference procedures were examined as robustness checks (Section 4.7) (Barr et al., 2013; Schielzeth & Forstmeier, 2009). The per-conversation slope analysis (Section 4.5) provides a complementary check by directly modeling conversation-level heterogeneity.

### 3.9. Topic Classification

We classify WildChat conversations into five categories based on keywords in the first user turn: coding (keywords: code, python, function, error, debug, javascript, html, css, sql, api, compile, syntax), creative writing (write, story, poem, essay, fiction, novel, script, dialogue, narrative), factual QA (explain, what is, how does, define, tell me about, describe), advice (help me, advice, recommend, suggest, should I), and other (no keyword match). This is a coarse heuristic classification for exploratory moderation analysis. The keyword lists are not mutually exclusive; in cases of overlap, we assign to the first matching category in the order listed above (coding takes priority). The advice category (22 conversations in the full sample) is small, and its results should be interpreted accordingly.

### 3.10. Turn Attrition

Because we restrict analysis to turns 1–10, but conversations vary in length, later turns have fewer observations. Table 1 reports the number of conversations contributing observations at each turn position.

Switchboard attrition is minimal (500 to 497 at turn 10) because conversations are typically long. WildChat attrition is negligible through turn 8 (1318 or more conversations contribute at each position) but increases at turns 9–10 (1313 and 1124, respectively), raising the possibility that conversations surviving to turn 10 are systematically different from those that end earlier. We address this with a survivorship check (reported in the Section 4).

## 4. Results

### 4.1. Descriptive Statistics

Table 2 presents the corpus-level statistics. WildChat conversations are shorter on average (36.7 turns in the full sample) than Switchboard (78.6 turns), reflecting the different interaction modalities, though the mean tokens per turn in WildChat (324 for users, 355 for assistants) dramatically exceed those in Switchboard (mean 18, median 11). The total volume of text analyzed per conversation is therefore much larger in WildChat despite fewer turns.

### 4.2. Within-Versus-Between Dissociation

Table 3 reports the unidirectional (user-side) multilevel model results, retained for comparison with the Switchboard baseline and syntactic similarity. The symmetric bidirectional analysis (Table 4) is the primary analysis.

**WildChat GPT-4o.** In the full sample of 1319 conversations (25,996 observations), within-conversation function word similarity is significantly higher than between-conversation similarity ($\beta_{1}=+0.036$, $p<10^{-22}$), indicating that users’ function word patterns are more similar to their own AI interlocutor’s patterns than to random AI turns. The between-conversation baseline is flat across turns ($\beta_{2}=-0.00007$, p=0.96), confirming that the baseline does not drift and that any change in the within-versus-between gap reflects within-conversation dynamics. The interaction term is marginally significant ($\beta_{3}=+0.002$, p=0.056), providing suggestive but not conclusive evidence that this similarity gap widens over turns in the full sample.

**Figure 1:**
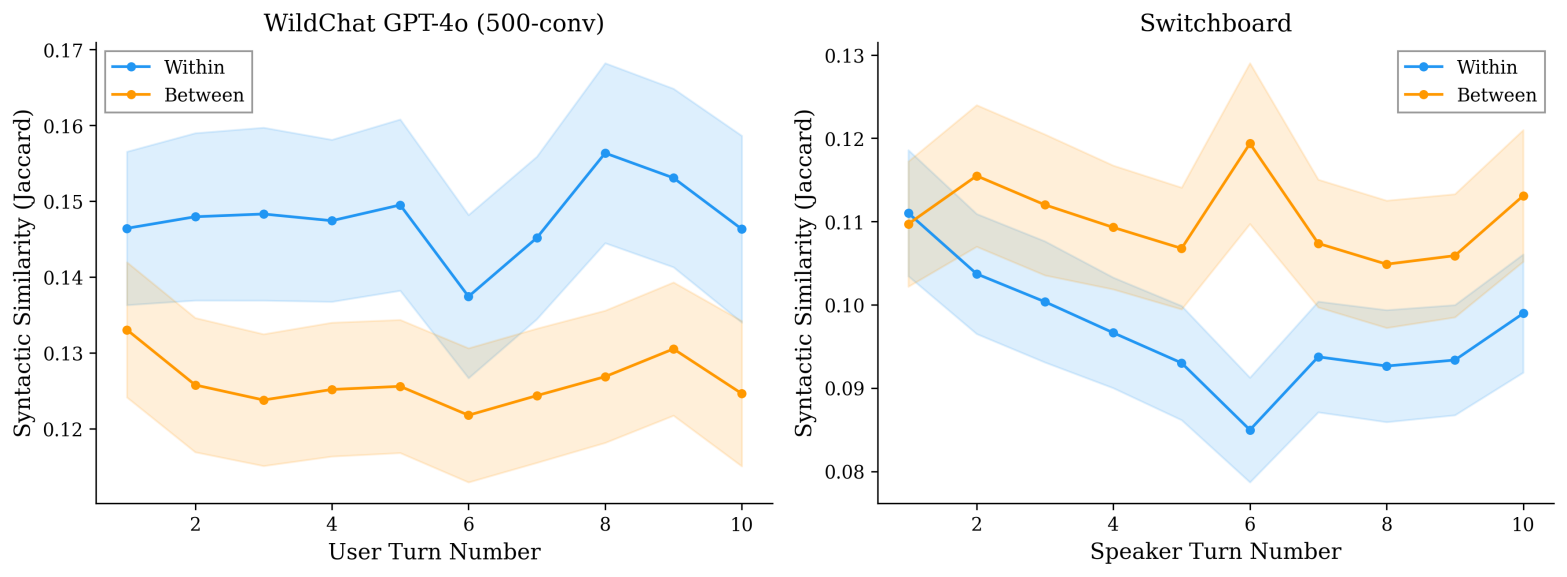
Syntactic Jaccard similarity trajectories (turns 1–10). (**Left**): WildChat GPT-4o (500-conv). (**Right**): Switchboard. Shaded: 95% CIs.

In the 500-conversation subsample with syntactic parsing (9852 observations), function word results are consistent ($\beta_{1}=+0.038$, p<0.001; $\beta_{3}=+0.004$, p=0.084). Syntactic similarity also shows a significant positive static effect ($\beta_{1}=+0.022$, p<0.001) with a non-significant interaction ($\beta_{3}=+0.001$, p=0.334). Figure 1 presents the syntactic similarity trajectories. The random effects for the 500-conversation function word model yield a conversation-level variance of $\sigma_{u}^{2}=0.048$ and residual variance of $\sigma_{e}^{2}=0.088$, providing an intraclass correlation of ICC = 0.35. This indicates that 35% of the variance in similarity scores is between conversations, reflecting substantial heterogeneity in baseline similarity levels across conversations (likely driven by topic and user differences).

**Switchboard.** Within-conversation function word similarity is significantly lower than between-conversation similarity ($\beta_{1}=-0.031$, p<0.001), and this gap shows a marginal widening over turns ($\beta_{3}=-0.003$, p=0.055). The between-conversation baseline is flat ($\beta_{2}=-0.0002$, p=0.88). Syntactic similarity shows a similar pattern ($\beta_{1}=-0.014$, p<0.001; $\beta_{3}=-0.001$, p=0.076). The per-conversation slope analysis (Section 4.5) confirms this divergence trend.

The random effects for the Switchboard function word model yield $\sigma_{u}^{2}=0.003$ and $\sigma_{e}^{2}=0.095$, giving ICC = 0.03. This near-zero ICC indicates that almost all variance is within-conversation: because each Switchboard conversation involves a unique speaker pair, the between-conversation random intercept captures idiosyncratic dyad effects that do not cluster systematically. The contrast with WildChat’s ICC of 0.35 follows from the interlocutor-homogeneity asymmetry: all WildChat conversations involve the same system (GPT-4o), so conversations that happen to share topic or user style produce stable between-conversation similarity differences that inflate the random-intercept variance.

**Figure 2:**
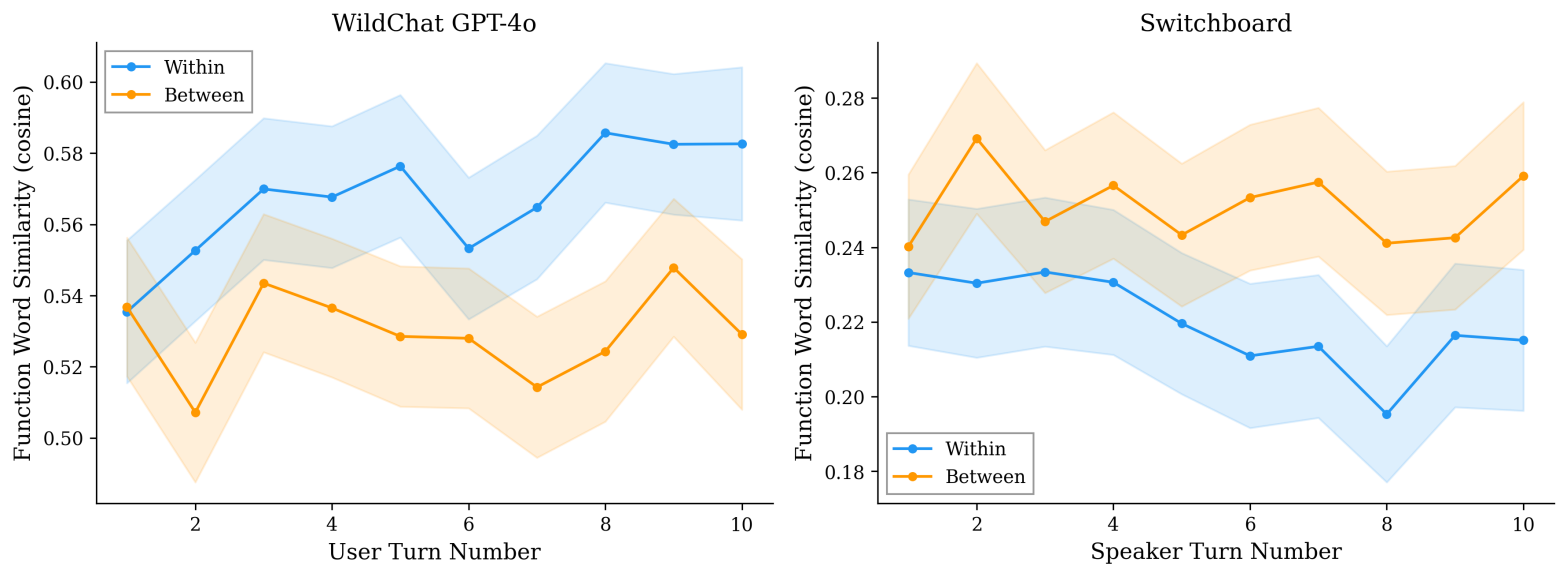
Function word cosine similarity trajectories (turns 1–10). (**Left**): WildChat GPT-4o. (**Right**): Switchboard. Shaded: 95% CIs.

Figure 2 visualizes the function word similarity trajectories for both datasets. The x-axis label “User Turn Number” is 1-indexed within the eligible range (N≥2), so position 1 corresponds to the second user message in each conversation, compared against the first assistant response.

The between-conversation baseline provides a critical validity check. The $\beta_{2}$ coefficient in Equation (1) tests whether this baseline drifts over turns. For WildChat (full sample), $\beta_{2}=-0.00007$ (p=0.96); for Switchboard, $\beta_{2}=-0.0002$ (p=0.88). Both are effectively zero, confirming that any change in the within-versus-between gap reflects within-conversation dynamics.

### 4.3. Bidirectional Comparison: User-Side vs. Model-Side

The unidirectional results in Table 3 use the original analysis pipeline (Section 3.2). The symmetric bidirectional analysis below uses the independently implemented pipeline on the full 1319-conversation sample. The user-side $\beta_{3}$ differs between the two tables (p=0.084 in the 500-conversation subsample vs. p=0.003 in the full sample) because of both the difference in sample size (9852 vs. 25,976 observations) and the independent between-conversation sampling. The full-sample bidirectional analysis is the primary analysis.

Table 4 reports the symmetric within-versus-between dissociation for both directions in the full WildChat GPT-4o sample (1319 conversations), with both directions using the harmonized turn window (turns 1–10).

**Figure 3:**
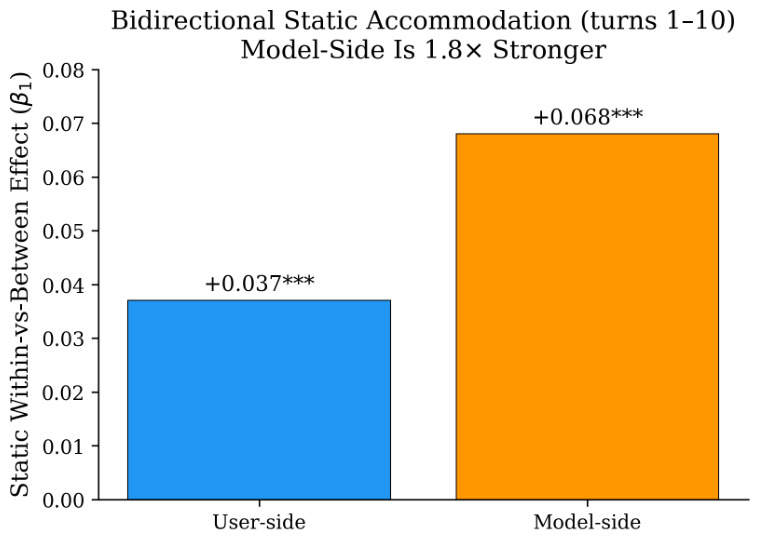
Static effect ($\beta_{1}$) by direction (turns 1–10, N=1319). *** p<0.001.

**Static effect ($\beta_{1}$).** The model-side static effect is 1.8× stronger than the user-side ($\beta_{1}=+0.068$ vs. +0.037, both p<0.001). GPT-4o’s function word patterns are substantially more similar to its own user than to random users, confirming the strong model-side accommodation documented by Blevins et al. (2026). A substantial portion of the within-versus-between gap in any unidirectional user-side analysis therefore reflects model-side accommodation. Figure 3 visualizes this asymmetry.

**Progressive dynamics ($\beta_{3}$).** Users show significant progressive convergence ($\beta_{3}=+0.004$, p=0.003): the within-versus-between gap widens over turns on the user side, with a flat between-conversation baseline ($\beta_{2}=+0.0003$, p=0.72). The model shows no progressive change ($\beta_{3}=+0.0002$, p=0.90): GPT-4o’s function word similarity to its own user, relative to the between-conversation baseline, is stable across turns 1–10. The model-side between-conversation baseline shows a small upward drift that does not reach significance ($\beta_{2}=+0.002$, p=0.12). The progressive signal is thus unidirectional: despite the model’s stronger static effect, the over-turn trajectory is driven entirely by the user side.

**Temporal dynamics.** The two sides exhibit qualitatively different temporal patterns. On the model side, the within-versus-between gap at turn 0 (the assistant’s first response, conditioned on the user’s opening message alone) is 0.171, approximately 2.5 times the mean gap across turns 1–10 (0.068). From turn 1 onward, the gap is stable ($\beta_{3}=+0.0002$, p=0.90). The model’s adaptation is front-loaded: a strong initial response to the user’s first message, followed by stabilization at a constant level of conversation-specific similarity. On the user side, the gap starts lower and widens gradually ($\beta_{3}=+0.004$, p=0.003), with the progressive convergence concentrated in interpersonal pronoun categories. When the model-side regression is fitted across turns 0–10, the decline from the turn-0 peak to the steady-state plateau produces a significantly negative $\beta_{3}$ (−0.005, p<0.001; category-level results in Appendix I), but this reflects stabilization after initial adaptation, not progressive divergence. The harmonized window (turns 1–10) is used for direct user-vs-model $\beta_{3}$ comparison because turn 0 has no user-side counterpart (the first user message has no preceding assistant turn).

### 4.4. Category-Level Decomposition of $\beta_{3}$

Decomposing $\beta_{3}$ across the 10 function word categories by fitting separate within-versus-between models for each category’s similarity reveals a structural dissociation (Table 5).

User-side progressive convergence is driven exclusively by interpersonal markers: second-person pronouns ($\beta_{3}=+0.00079$, p<0.001) and first-person singular pronouns ($\beta_{3}=+0.00063$, p<0.001), both surviving Bonferroni correction (α/20=0.0025). Topic-related categories (articles, prepositions, conjunctions) show no significant user-side progressive change. On the model side, no category shows a significant progressive effect after Bonferroni correction; the uncorrected trends in conjunctions (p=0.044) and first-person singular pronouns (p=0.021) are positive (convergence toward the user), not negative. The model side does not progressively diverge in any function word category at the harmonized window.

**Figure 4:**
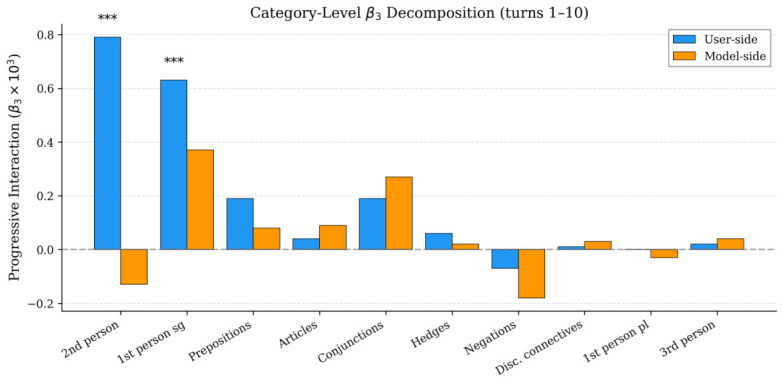
Category-level $\beta_{3}$ (turns 1–10, N=1319). *** p<0.001. Dashed line: zero.

The progressive user-side signal reflects shifting pronoun usage (users increasingly adopting the model’s low-first-person, moderate-second-person pattern), not topic-specific vocabulary change. The cumulative effect sizes for the two significant pronoun categories are d=0.22 (second-person pronouns) and d=0.14 (first-person singular), computed as $d=\left(\beta_{3}\times 9\right)/{\mathrm{SD}}_{\mathrm{pooled}}$ over the nine-turn trajectory. These fall within the range of effect sizes reported for function word style matching in naturalistic conversational settings (Bierstetel et al., 2020; Dideriksen et al., 2023). Figure 4 visualizes this pattern.

#### First-Person Singular Pronoun Decline

First-person singular pronoun usage declines significantly across turns in WildChat GPT-4o ($M_{\mathrm{slope}}=-0.0006$, t(1318)=−4.92, p<0.001; see Figure A5 in Appendix G). A comparable decline occurs in Switchboard ($M_{\mathrm{slope}}=-0.0010$, t(499)=−4.49, p<0.001; Figure A6), suggesting this is a general conversational warmup effect (speakers become less self-referential as conversations develop) rather than an LLM-specific accommodation pattern.

This shared pronoun decline is informative for interpreting the convergence–divergence asymmetry. In WildChat, declining first-person singular usage moves users toward GPT-4o’s output style (which uses relatively few first-person singular pronouns), contributing to the convergence signal. In Switchboard, the same pronoun decline moves both speakers in the same direction (both reduce self-reference), which does not produce convergence because both interlocutors are changing in parallel. The same underlying process (conversational warmup reducing self-reference) manifests differently in the within-versus-between design depending on whether the interlocutor is also changing (Switchboard) or is relatively stable (WildChat, where GPT-4o’s output style is consistent across turns).

### 4.5. Per-Conversation Convergence Slopes

The per-conversation slope analysis addresses a complementary question. $\beta_{3}$ tests whether the within-versus-between gap changes over turns; per-conversation slopes test whether within-conversation similarity increases over turns for individual conversations, without reference to a between-conversation baseline.

**Figure 5:**
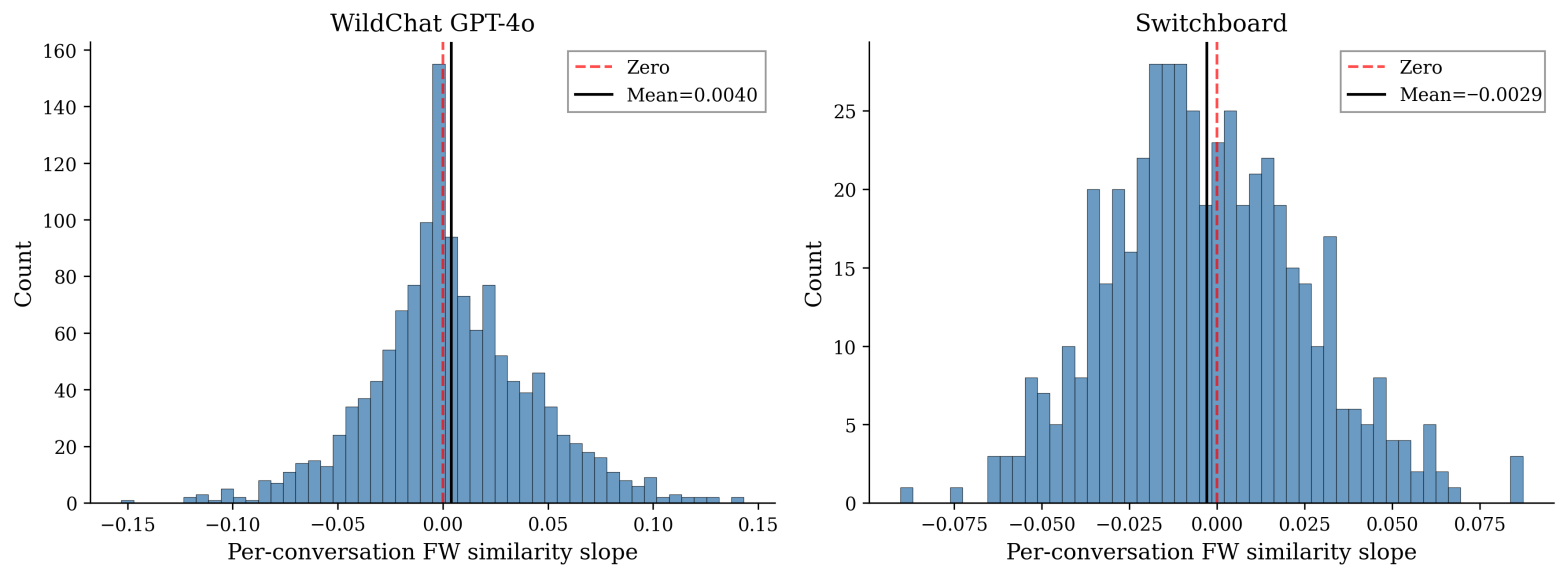
Per-conversation function word similarity slopes (turns 1–10). (**Left**): WildChat GPT-4o (full sample, N=1319). (**Right**): Switchboard (N=500). Black line: mean. Red dashed: zero.

In the full WildChat GPT-4o sample (1319 conversations), the mean per-conversation slope is significantly positive (M=+0.0040, t(1318)=3.78, p<0.001), with 50.2% of conversations showing positive slopes. In Switchboard, the mean slope is significantly negative (M=−0.0029, t(499)=−2.30, p=0.022), with 43.6% positive slopes. Figure 5 shows the slope distributions for the full WildChat sample and the Switchboard sample.

The proportion of positive slopes (50.2%) indicates that convergence and divergence are distributed almost evenly across conversations, with a small but reliable tilt toward convergence in the aggregate, consistent with the small effect size (d=0.09) and substantial individual variability. Because the between-conversation baseline is flat ($\beta_{2}\approx 0$), the per-conversation slopes and the multilevel $\beta_{3}$ are approximately aligned.

### 4.6. Survivorship Check

Because WildChat shows turn attrition at later positions (1319 to 1124 conversations at turn 10; Table 1), we check whether conversations that survive to later turns have systematically different similarity levels. We correlate each conversation’s total number of turns with its mean within-conversation function word similarity across the full sample (N=1319). The correlation is negligible (r=+0.011, p=0.68), providing no evidence of survivorship bias: conversations that last longer do not have higher (or lower) baseline similarity. This does not rule out all forms of selection bias, but it addresses the most direct concern that the convergence signal is driven by higher-similarity conversations persisting longer.

### 4.7. Robustness of the Progressive Convergence Effect

#### 4.7.1. Random-Effects Specification

To address concerns that random-intercept-only models may produce anti-conservative standard errors for $\beta_{3}$ (Barr et al., 2013), we fitted random-slope specifications, adding slopes for IsWithin, Turn, or both. All three converge with boundary warnings (MLE near the edge of the parameter space). Random slope variances are small relative to the intercept variance (IsWithin slope variance ≈0.0014–0.0017; Turn slope variance ≈0.0006; intercept variance ≈0.038–0.045). The $\beta_{3}$ estimate is unchanged ($\beta_{3}=0.00382$, p=0.003 in all cases). A pairs cluster bootstrap (1000 conversation-level resamples) yields a 95% BCa CI of [0.0021,0.0057], excluding zero. OLS with cluster-robust standard errors confirms significance ($\beta_{3}=0.00382$, p<0.001).

#### 4.7.2. Between-Conversation Sampling Sensitivity

Because each within-conversation observation is paired with a single randomly sampled between-conversation partner, the $\beta_{3}$ estimate depends on this sampling. We repeated the full analysis across 10 different random seeds. The user-side aggregate $\beta_{3}$ is positive and significant in all 10 runs (range [+0.0028,+0.0047], mean +0.0037, p<0.05 in 10/10). At the category level, second-person pronoun $\beta_{3}$ is significant in 10/10 runs and first-person singular $\beta_{3}$ in 10/10 runs. No other category reaches significance in more than 1 of 10 runs. On the model side, the aggregate $\beta_{3}$ is non-significant in all 10 runs (range [−0.0014,+0.0009], 0/10 at p<0.05), confirming that the model side shows no progressive change at the harmonized window.

#### 4.7.3. Analysis Window Sensitivity

The harmonized window (turns 1–10) was adopted to ensure comparability across directions (Section 3.2). The model-side turn-0 observation reported in Section 4 shows that including or excluding this single turn position produces qualitatively different model-side $\beta_{3}$ estimates (from −0.005, p<0.001 at turns 0–10 to +0.0002, p=0.90 at turns 1–10). The user-side $\beta_{3}$ is stable across windows: +0.004 (p=0.003) at turns 1–10 and +0.004 (p=0.009) at turns 1–9, becoming non-significant only at turns 2–10 (+0.002, p=0.19), where statistical power is reduced. The Switchboard per-conversation slope (Section 4.5) remains significantly negative at all windows tested.

### 4.8. Topic Moderation

Table 6 reports user-side convergence by topic category in the full WildChat sample (1319 conversations).

**Figure 6:**
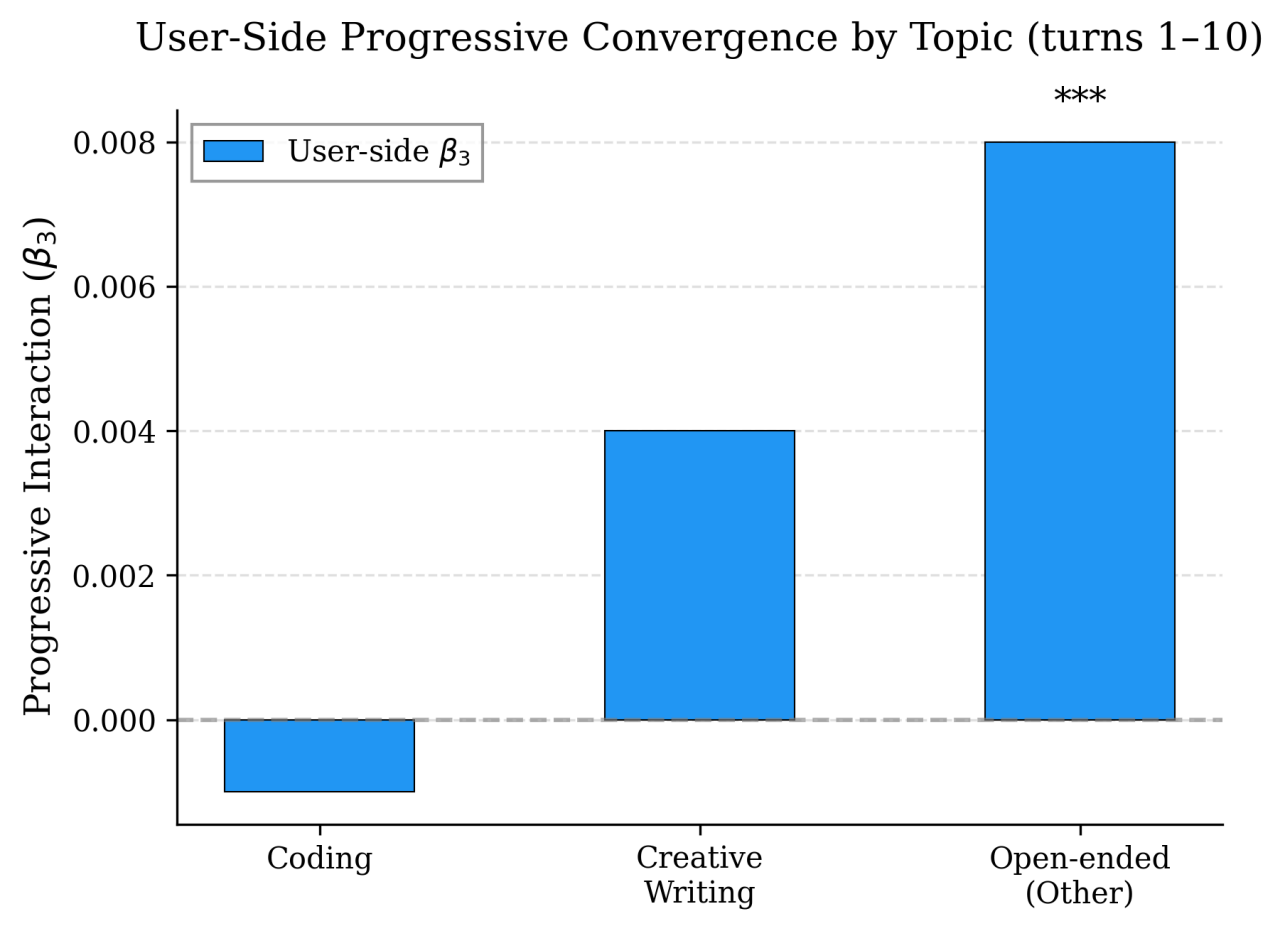
User-side $\beta_{3}$ by topic category (turns 1–10). *** p<0.001.

Task-constrained conversations (coding: 465, factual QA: 54) show strong positive static effects ($\beta_{1}=+0.055$ for both) but no progressive convergence ($\beta_{3}\approx 0$). The high $\beta_{1}$ reflects topic-driven similarity. Creative writing (152) follows the same pattern ($\beta_{1}=+0.061$, no significant $\beta_{3}$). The residual category (626 conversations lacking keyword matches, comprising translation requests, brainstorming, casual exchanges, role-play, and mixed-intent multi-step requests) shows a weaker static effect ($\beta_{1}=+0.018$, p=0.002) but the strongest progressive convergence ($\beta_{3}=+0.008$, p<0.001). Low static similarity combined with significant progressive change is consistent with accommodation operating when task constraints do not already fix function word usage. Figure 6 visualizes this pattern.

### 4.9. Cross-Model Consistency

**Figure A4:**
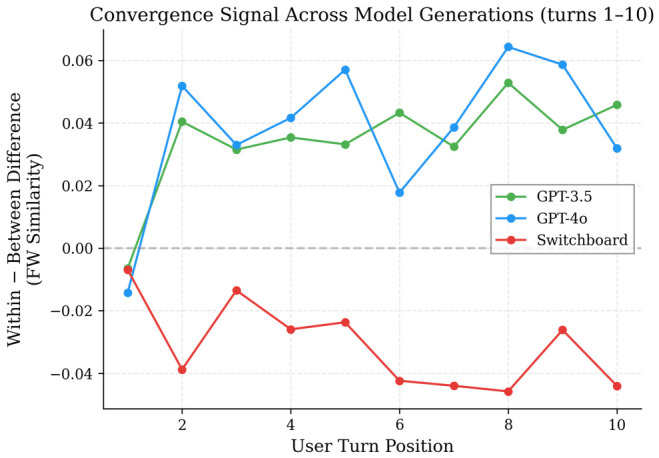
Within-minus-between function word similarity difference across turns and model generations. Turn position uses the same 1-indexed convention as Figure 2. Dashed line: zero.

**Figure A5:**
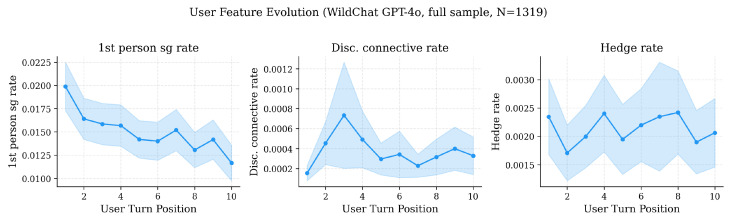
User feature evolution across turns in WildChat GPT-4o (full sample, N=1319).

**Figure A6:**
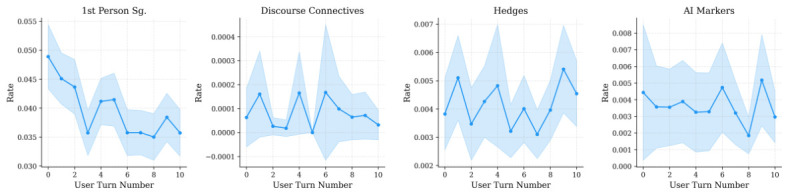
User feature evolution across turns in Switchboard.

The user-side convergence pattern is consistent across model generations: GPT-3.5 conversations (499 conversations, 9760 observations) show a positive static effect ($\beta_{1}=+0.034$, p<0.001) and a non-significant convergence trend ($\beta_{3}=+0.004$, p=0.109). The directional consistency despite user population confounds across collection periods suggests the static effect is robust. Figure A4 in Appendix F visualizes the cross-model comparison.

## 5. Discussion

### 5.1. Progressive Interpersonal Convergence in Human–AI Conversation

The 1.8× model-side advantage in the static effect ($\beta_{1}=+0.068$ vs. +0.037) confirms that model-side adaptation contributes a substantially larger within-versus-between gap than user-side adaptation, consistent with Blevins et al. (2026). Category-level decomposition of $\beta_{1}$ shows that the static effect is concentrated in prepositions ($\beta_{1}=+0.010$ user, +0.014 model) and articles ($\beta_{1}=+0.009$ user, +0.013 model), both topic-related structural categories, and is largest in task-constrained categories (coding, factual QA). A unidirectional user-side analysis conflates these two sources.

The progressive interaction ($\beta_{3}$) separates from the static effect along two dimensions: direction and category. Users show significant progressive convergence ($\beta_{3}=+0.004$, p=0.003), while the model shows no progressive change ($\beta_{3}\approx 0$, p=0.90). The category-level decomposition (Table 5) locates this progressive signal exclusively in interpersonal markers: second-person pronouns (p<0.001) and first-person singular pronouns (p<0.001), with zero progressive change in topic-driven categories. Users’ pronoun usage patterns shift toward the model’s characteristic low-first-person, moderate-second-person register as conversations develop.

The first-person singular pronoun decline is itself a general conversational warmup effect shared with Switchboard (Section 4), but the within-versus-between design captures its differential contribution: in WildChat, the user’s pronoun decline toward GPT-4o’s stable register produces increasing conversation-specific similarity, while in Switchboard, parallel declines by both speakers produce no such widening gap.

The category-specificity of this signal provides the main evidence for distinguishing among candidate mechanisms. A pure automatic priming account (Pickering & Garrod, 2004) predicts convergence across all function word categories to which the user is exposed: if the mechanism is non-selective repetition of recently encountered structures, the effect should appear in articles, prepositions, and conjunctions as well as in pronouns, since all of these categories are present in the model’s output at comparable rates. The data do not follow this prediction. Progressive convergence is restricted to second-person and first-person singular pronouns, the two categories that Pennebaker et al. (2003) identified as primary linguistic markers of self-referential focus and interpersonal orientation, and that correlate with Big Five personality dimensions (neuroticism with first-person singular frequency, agreeableness with second-person frequency, per Ireland et al. 2011; Pennebaker et al. 2003; Yarkoni 2010). The restriction of progressive convergence to these categories, with no progressive change in structural categories, indicates that the signal operates selectively on the self-presentational dimensions of language use. This argument addresses the simplest form of the priming hypothesis, in which alignment is non-selective across exposed categories. A more nuanced priming account in which socially salient categories receive stronger alignment cannot be excluded by the present data.

The task-context pattern strengthens this interpretation. In coding conversations, where the strong static effect ($\beta_{1}=+0.055$) confirms substantial function word similarity at baseline, progressive convergence is absent ($\beta_{3}\approx 0$). In the residual non-keyword-matched category (labeled “open-ended,” comprising translation, brainstorming, casual exchanges, role-play, and mixed-intent requests), where the static effect is weakest ($\beta_{1}=+0.018$), progressive convergence is strongest ($\beta_{3}=+0.008$, p<0.001). Factual QA ($\beta_{1}=+0.055$) likewise shows no progressive trend. A ceiling-effect account (high baseline similarity leaves less room for progressive change) does not fully explain this pattern, because creative writing conversations have the highest baseline ($\beta_{1}=+0.061$) yet show a non-significant positive trend ($\beta_{3}=+0.004$, p=0.25). The three-way pattern (high static with no progression in both coding and factual QA, low static with strong progression in open-ended) is more consistent with task constraints limiting self-presentational flexibility than with a proximity-to-ceiling artifact. The aggregate effect size (d=0.09) reflects the 10-dimensional cosine measure, which weights all function word categories equally and dilutes a domain-specific effect whose category-level magnitude (d=0.22 for second-person pronouns) falls within the range of LSM effects documented in human–human interaction (Bierstetel et al., 2020; Dideriksen et al., 2023).

This pronoun-level convergence is consistent with the self-concept alignment effects documented by Li et al. (2026) (Section 2), in that the pronoun dimensions on which users converge are the same dimensions through which self-presentational orientation is linguistically instantiated (Pennebaker et al., 2003; Yarkoni, 2010). The progressive increase in second-person pronoun similarity is also compatible with a relational-engagement reading, as documented in recent work on AI attachment (Ho et al., 2025; Kasturiratna & Hartanto, 2025). The category-specificity evidence is consistent with both accounts, as both predict convergence on interpersonal pronoun categories. Disentangling the two requires direct relational and personality measurements alongside the linguistic measures.

The symmetric design resolves the identification problem and reveals two distinct temporal dynamics. The model’s adaptation is front-loaded: a strong initial response to the user’s first message (within-versus-between gap of 0.171 at turn 0, settling to a stable 0.068 across turns 1–10), followed by no further progressive change within the harmonized window. The user starts from a lower baseline and converges gradually on interpersonal pronoun dimensions. These two patterns correspond to different generative processes: the model’s front-loaded adaptation reflects conditional text generation responding to available conversational context, while the user’s gradual convergence operates selectively on self-presentational dimensions. The progressive user-side signal cannot be attributed to increasing model-side accommodation because the model side is stable once the initial adaptation has occurred. If within-conversation pronoun shifts persist across interactions, they could contribute to the population-level homogenization documented by Liang et al. (2025), Sourati et al. (2026), and Padmakumar and He (2024). Whether such persistence occurs is an open empirical question requiring longitudinal data.

### 5.2. The Switchboard Comparison

The Switchboard per-conversation slope analysis (Section 4.5) is significantly negative (p=0.022), though the multilevel interaction term is marginal ($\beta_{3}=-0.003$, p=0.055). This pattern is consistent with Healey et al. (2014) and with the alignment-plus-complementarity framework (Fusaroli et al., 2014), in which speakers who establish common ground (Clark, 1996) differentiate into complementary communicative roles, producing decreasing function word similarity even as they align at other levels (Reitter & Moore, 2014; Xu & Reitter, 2016a). The progressive component is modest relative to the large static effect ($\beta_{1}=-0.031$).

The contrast between corpora remains structurally informative despite this weaker progressive signal. In Switchboard, speakers’ function word similarity declines over turns. In WildChat, user-side similarity increases. User-side progressive convergence in open-ended WildChat conversations has no Switchboard analogue. Whether this contrast reflects the stylistically consistent, non-competing nature of the AI interlocutor or the modality and interlocutor-homogeneity differences between the corpora cannot be fully determined from these data. A written human–human baseline is needed to disambiguate modality from interlocutor effects.

### 5.3. Limitations

Three structural limitations constrain interpretation. The WildChat–Switchboard comparison involves a written-spoken modality confound (Biber, 1991; Chafe, 1982) and an interlocutor-homogeneity asymmetry (one system vs. diverse speaker pairs). The convergence–divergence contrast cannot be causally attributed to interlocutor type without a written human–human baseline. WildChat users self-selected into multi-turn conversations, introducing selection effects. The observational design cannot establish causal direction: experimental manipulation of model style mid-conversation would be needed to confirm that the progressive user-side signal reflects accommodation. The random-intercept-only models were supplemented by random-slope specifications and cluster-bootstrap inference as robustness checks (Section 4.7), all of which confirm $\beta_{3}$ significance. The keyword-based topic classification is heuristic, and individual-difference predictors (prior LLM experience, Big Five personality traits, linguistic background) remain unexamined. The last gap is consequential for the personality implications: demonstrating that users with different personality profiles show different accommodation trajectories would strengthen the link between the linguistic convergence documented here and personality-level processes. The model-side turn-0 observation (Section 4) shows that including or excluding the assistant’s first response changes the model-side $\beta_{3}$ from −0.005 (p<0.001) to +0.0002 (p=0.90), a qualitative reversal driven by a single turn position per conversation. The harmonized window (turns 1–10) ensures comparability across directions, but this sensitivity means that the model-side progressive null is conditional on the analysis window. The atypically large turn-0 within-versus-between gap may reflect the model’s strong initial conditioning on the user’s opening message, possibly amplified by user-configured system prompts or memory settings in the WildChat data source, which are not observable in the corpus metadata.

## 6. Conclusions

A symmetric within-versus-between dissociation design applied to 1319 WildChat GPT-4o conversations reveals two distinct temporal dynamics: the model’s adaptation is front-loaded, with strong initial accommodation followed by stabilization, while users converge gradually on interpersonal pronoun dimensions across subsequent turns ($\beta_{3}=+0.004$, p=0.003). The model-side static effect is 1.8× stronger than the user-side ($\beta_{1}=+0.068$ vs. +0.037), and user-side convergence is driven exclusively by second-person and first-person singular pronouns with no progressive change in topic-related categories. This progressive convergence has no counterpart in the Switchboard human–human baseline, where per-conversation similarity slopes are significantly negative (p=0.022) (Fusaroli et al., 2014; Healey et al., 2014).

These pronoun categories are among the primary linguistic markers through which self-referential focus and interpersonal orientation manifest in natural language (Pennebaker et al., 2003). The present findings identify a candidate linguistic pathway that complements the self-concept alignment documented by Li et al. (2026): users’ pronoun usage converges selectively on these interpersonal dimensions and does so progressively over the conversation. The concentration of this convergence in open-ended conversations (where interpersonal orientation is unconstrained) and its absence in task-constrained conversations (where function word usage is dictated by the task) reinforce the interpretation that the signal is selective to interpersonally expressive dimensions of language use.

Three empirical priorities follow: a written human–human baseline to disambiguate modality from interlocutor effects, experimental manipulation of model style to establish causal direction, and longitudinal analysis to determine whether within-conversation pronoun shifts persist in users’ subsequent communication. Population-level evidence that LLM interaction is reshaping human expression continues to accumulate (Liang et al., 2025; Sourati et al., 2026; Yakura et al., 2024). The progressive interpersonal convergence documented here identifies a candidate within-conversation mechanism and the specific pronoun dimensions on which it may operate.   

## Figures and Tables

**Table 1:** Turn attrition: number of conversations contributing observations at each turn position.

	T1	T2	T3	T4	T5	T6	T7	T8	T9	T10
WildChat	1318	1319	1319	1319	1319	1319	1319	1319	1313	1124
Switchboard	500	500	500	500	500	500	499	499	498	497

**Table 2:** Descriptive statistics (unidirectional pipeline).

	WildChat GPT-4o	WildChat GPT-4o	Switchboard
	(Full, FW only)	(Subsample, +Syntax)	
Conversations	1319	500	500
Total turns	48,445	18,634	39,310
Avg. turns/conv.	36.7	37.3	78.6
Observations (turns 1–10)	25,996	9852	19,952
Mean tokens/turn (user)	324	324	18
Mean tokens/turn (partner)	355	355	18

**Table 3:** Unidirectional user-side results (original pipeline). ^†^ p<0.10, *** p<0.001.

Dataset	Indicator	$\beta_{1}$	$\beta_{2}$	$\beta_{3}$	$\sigma_{u}^{2}$	$\sigma_{e}^{2}$	ICC	*d*
WildChat (full)	FW sim.	+0.036 ***	−0.00007	$+0.002^{\;†}$	—	—	—	+0.09
WildChat (sub.)	FW sim.	+0.038 ***	—	$+0.004^{\;†}$	0.048	0.088	0.35	+0.10
WildChat (sub.)	Syntactic	+0.022 ***	—	+0.001	—	—	—	—
Switchboard	FW sim.	−0.031 ***	−0.0002	$-0.003^{\;†}$	0.003	0.095	0.03	−0.11
Switchboard	Syntactic	−0.014 ***	—	$-0.001^{\;†}$	—	—	—	—

**Table 4:** Bidirectional results (harmonized turns 1–10, N=1319). ** p<0.01, *** p<0.001.

Direction	$N_{\mathrm{obs}}$	$\beta_{1}$	$\beta_{2}$	$\beta_{3}$	$\sigma_{u}^{2}$	$\sigma_{e}^{2}$	ICC
User-side	25,976	+0.037 ***	+0.0003	+0.004 **	0.045	0.089	0.34
Model-side	26,000	+0.068 ***	+0.002	+0.0002	0.023	0.113	0.18
Ratio	—	1.8×	—	—	—	—	—

**Table 5:** Category-level $\beta_{3}$ by direction (turns 1–10, N=1319). Values $\times 10^{3}$. * p<0.05, *** p<0.001.

Category	User $\beta_{3}$	*p*	Model $\beta_{3}$	*p*
Second-person pronouns	+0.79 ***	<0.001	−0.13	0.28
First-person sg pronouns	+0.63 ***	<0.001	+0.37 *	0.021
Prepositions	+0.19	0.38	+0.08	0.73
Articles	+0.04	0.83	+0.09	0.65
Conjunctions	+0.19	0.18	+0.27 *	0.044
Negations	−0.07	0.63	−0.18	0.26
Hedges	+0.06	0.28	+0.02	0.70
Discourse connectives	+0.01	0.65	+0.03	0.16
First-person pl pronouns	+0.00	0.98	−0.03	0.65
Third-person pronouns	+0.02	0.85	+0.04	0.61

**Table 6:** User-side convergence by topic category (WildChat GPT-4o, full sample). *N*: number of conversations per category.

Topic	*N*	$\beta_{1}$	$p(\beta_{1})$	$\beta_{3}$	$p(\beta_{3})$
Coding	465	+0.055	<0.001	−0.001	0.80
Creative writing	152	+0.061	<0.001	+0.004	0.25
Factual QA	54	+0.055	0.002	−0.001	0.90
Advice	22	+0.007	0.81	−0.002	0.85
Other	626	+0.018	0.002	+0.008	<0.001

**Figure A1:**
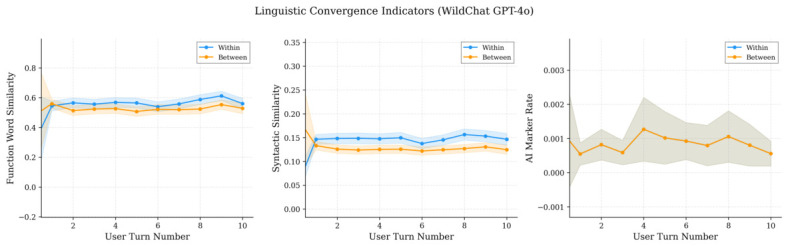
Three convergence indicators for WildChat GPT-4o (500-conversation subsample). (**Left**): function word similarity. (**Center**): syntactic similarity. (**Right**): AI marker rate (see text for interpretive caveat). Shaded: 95% CIs.

**Figure A2:**
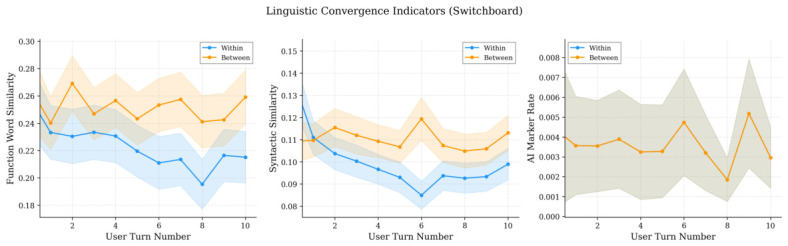
Three convergence indicators for Switchboard. (**Left**): function word similarity. (**Center**): syntactic similarity. (**Right**): AI marker rate. Shaded: 95% CIs. The Within/Between legend in the Right panel is not applicable to AI marker rate; see Appendix D.

**Figure A3:**
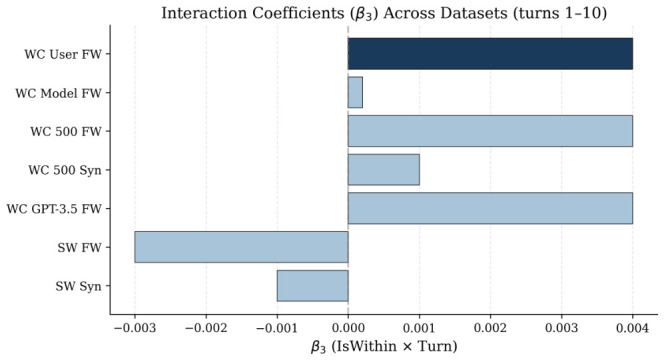
Interaction coefficients ($\beta_{3}$: IsWithin × Turn) across datasets and indicators. Darker bars: p<0.05.

**Figure A7:**
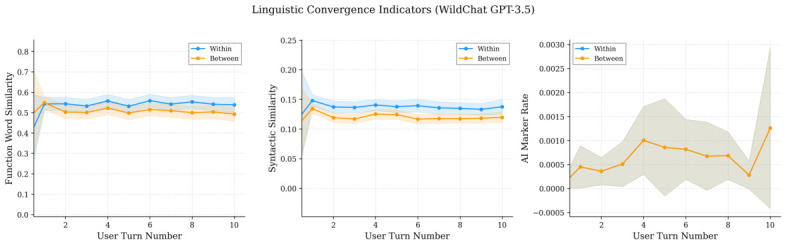
Three convergence indicators for WildChat GPT-3.5 (499 conversations). (**Left**): function word similarity. (**Center**): syntactic similarity. (**Right**): AI marker rate. The Within/Between legend in the Right panel is not applicable to AI marker rate; see Appendix D.

**Figure A8:**
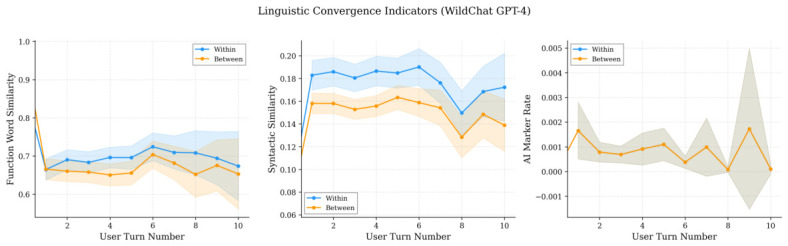
Three convergence indicators for WildChat GPT-4 (22 conversations; sample is too small for reliable inference). (**Left**): function word similarity. (**Center**): syntactic similarity. (**Right**): AI marker rate. The Within/Between legend in the Right panel is not applicable to AI marker rate; see Appendix D.

## Data Availability

The WildChat dataset is publicly available at https://huggingface.co/datasets/allenai/WildChat (accessed on 1 March 2026). The Switchboard corpus is available through the Linguistic Data Consortium. Analysis code and processed data will be made available upon publication.

## Supplementary Materials

The following supporting information can be downloaded at: https://www.mdpi.com/article/10.3390/bs16050720/s1. Dataset S1: Analysis code, model results, raw similarity data, and trajectory data.

## Appendix A. Function Word Categories

We define 10 function word categories based on the LIWC framework (Tausczik & Pennebaker, 2010), using open-source word lists rather than the proprietary LIWC lexicon:

- First-person singular: I, me, my, mine, myself
- First-person plural: we, us, our, ours, ourselves
- Second-person: you, your, yours, yourself, yourselves
- Third-person: he, him, his, she, her, they, them, their, etc.
- Discourse connectives: however, therefore, moreover, furthermore, additionally, nevertheless, consequently, etc.
- Hedges: maybe, perhaps, possibly, probably, likely, might, could, somewhat, etc.
- Articles: a, an, the
- Prepositions: in, on, at, to, for, with, by, from, of, about, into, through, etc.
- Conjunctions: and, but, or, because, although, while, if, when, since, unless, etc.
- Negations: no, not, never, neither, nobody, nothing, cannot, don’t, doesn’t, etc.

## Appendix B. AI-Characteristic Discourse Markers

We constructed a preliminary AI-characteristic marker lexicon through manual comparison of GPT-4 output patterns with general written English. Per-conversation slope analysis on this lexicon yielded a null result (M=0.00004, t(136)=0.37, p=0.714) due to floor effects (base rate <0.002 per token). We do not report this analysis in the main text because the lexicon lacks validation (no frequency threshold, no comparison against human-helper corpora, no inter-annotator agreement). A validated AI-marker lexicon is needed before lexical-level convergence can be assessed.

## Appendix C. Statistical Model Details

All models use statsmodels.mixedlm with REML estimation and Powell optimization. The Turn variable is centered at the sample mean (mean values: WildChat full sample 4.82, WildChat subsample 4.85, Switchboard 4.91). Random effects include a random intercept for conversation ID only. All random-intercept models converged without warnings. Random-slope specifications, examined as robustness checks (Section 4.7), converge with boundary warnings indicating that the MLE is near the edge of the parameter space; see Section 4.7 for the full comparison. Analyses are limited to turns 1–10. SpaCy version: 3.8.11; model: en_core_web_sm.

## Appendix D. Three-Indicator Panel Figures

Figure A1 and Figure A2 present three-indicator panels (function word similarity, syntactic similarity, and AI marker rate) for WildChat GPT-4o and Switchboard. For function word and syntactic similarity, the “Within” and “Between” lines represent the within-versus-between dissociation. For the AI marker rate, the two lines represent average user marker rates grouped by observation type; because marker rates are computed from user text alone rather than as a user-assistant similarity measure, the within-versus-between contrast is not interpretively meaningful for this indicator (see Section 4 for the appropriate per-conversation slope analysis).

## Appendix E. Effect Size Comparison

Figure A3 summarizes the interaction coefficients ($\beta_{3}$) across all datasets and indicators, providing a visual comparison of convergence (positive) versus divergence (negative) effects.

## Appendix F. Cross-Model Comparison Details

The cross-model analysis includes GPT-3.5 (499 conversations, 9760 observations) and GPT-4o (500-conversation subsample, 9852 observations). GPT-4 data was downloaded but produced only 22 qualifying conversations after English and multi-turn filtering, insufficient for reliable analysis; it is excluded from both statistical modeling and the cross-model figure. Figure A4 plots the within-minus-between difference across turns for GPT-3.5, GPT-4o, and Switchboard.

GPT-3.5 results: $\beta_{1}=+0.034$ (p<0.001), $\beta_{3}=+0.004$ (p=0.109). The consistency of the positive direction across model generations, despite user population confounds, is suggestive of a robust static effect, though the progressive component does not reach significance in either model subsample individually.

## Appendix G. User Feature Evolution Figures

Figure A5 and Figure A6 show the evolution of individual user-side linguistic features across turns, including first-person singular pronouns, discourse connectives, hedges, and AI marker rates.

## Appendix H. Additional Three-Indicator Panels

Figure A7 and Figure A8 present three-indicator panels for additional WildChat model subsamples.

## Appendix I. Model-Side Category-Level Results: Turns 0–10 and 1–10

When the model-side analysis window turns 0, the aggregate $\beta_{3}$ is significantly negative (−0.005, p<0.001), reflecting the decline from the large turn-0 within-versus-between gap (0.171) to the steady-state level (0.068). Table A1 decomposes this effect across the 10 function word categories. The negative $\beta_{3}$ is concentrated in structural categories (prepositions, articles), not interpersonal markers. Second-person and first-person singular pronouns show no model-side progressive change at either window. The sign reversals in structural categories between windows (e.g., prepositions shifting from −0.83 at turns 0–10 to +0.08 at turns 1–10) reflect the same turn-0 transition that drives the aggregate sign change: including turn 0 captures the model’s initial-conditioning effect, while the harmonized window captures only the post-stabilization regime.

**Table A1:** Model-side category-level $\beta_{3}$ at turns 0–10 (N=1319). Values $\times 10^{3}$. * p<0.05, ** p<0.01, *** p<0.001.

Category	Turns 0–10 $\beta_{3}$	*p*	Turns 1–10 $\beta_{3}$	*p*
Prepositions	−0.83 ***	<0.001	+0.08	0.73
Articles	−0.56 **	0.001	+0.09	0.65
Negations	−0.27 *	0.048	−0.18	0.26
Conjunctions	−0.23	0.051	+0.27 *	0.044
Hedges	−0.05	0.30	+0.02	0.70
First-person pl pronouns	−0.04	0.47	−0.03	0.65
Discourse connectives	+0.02	0.36	+0.03	0.16
Second-person pronouns	+0.01	0.96	−0.13	0.28
Third-person pronouns	+0.02	0.81	+0.04	0.61
First-person sg pronouns	+0.18	0.20	+0.37 *	0.021
